# Effects of Different Exercise Interventions on Cardiac Autonomic Control and Secondary Health Factors in Middle-Aged Adults: A Systematic Review

**DOI:** 10.3390/jcdd8080094

**Published:** 2021-08-05

**Authors:** Bernhard Grässler, Beatrice Thielmann, Irina Böckelmann, Anita Hökelmann

**Affiliations:** 1 Department of Sport Science, Faculty of Humanities, Otto von Guericke University, 39106 Magdeburg, Germany; anita.hoekelmann@ovgu.de; 2 Department of Occupational Medicine, Medical Faculty, Otto von Guericke University, 39120 Magdeburg, Germany; beatrice.thielmann@med.ovgu.de (B.T.); irina.boeckelmann@med.ovgu.de (I.B.)

**Keywords:** cardiac autonomic control, heart rate variability, autonomic nervous system, physical intervention, secondary health factors, healthy adults, middle age

## Abstract

This systematic review was conducted in accordance with the PRISMA guidelines to summarize the existing literature on the effects of different exercise interventions on cardiac autonomic control and secondary health factors. Resting heart rate variability (HRV) was used as indicator of cardiac autonomic control. Secondary factors were related to factors that contribute to cardiovascular health. Studies examining the effects of endurance, resistance, multimodal, or coordinative training interventions in healthy participants aged between 45 and 60 years old on average were considered. The methodological quality of the studies was examined using two assessment scales (TESTEX and STARD_HRV_). PROSPERO registration number: CRD42020206606. The literature review retrieved eight studies fulfilling all inclusion criteria. Cardiac autonomic control and cardiovascular health improved after endurance and multimodal interventions. Resistance training had no significant impact on HRV or any secondary health factor. Coordinative exercise interventions showed inconclusive results regarding HRV but showed significant improvements in secondary health factors. The quality assessment tools revealed some methodological and reporting deficits. Despite the small number of studies, we suggest endurance and multimodal interventions including aerobic exercises for the enhancement of cardiac autonomic control and the reduction of cardiovascular risk in middle-aged adults. Further studies need to be conducted to examine the long-term effects of exercise in the midlife period.

## 1. Introduction

Aging is associated with several physical impairments. Among them are decreasing parasympathetic and increasing sympathetic modulation, and impaired baroreflex sensitivity, leading to autonomic imbalance [1], increasing blood pressure [2], decreasing cardiorespiratory fitness, and decreasing muscular strength [3]. Age-related changes in autonomic modulation are caused by the reduced elasticity of barosensory vessels [4], increased arterial stiffness [5], changes in efferent neuronal conduction [6], and decreased baroreceptor sensitivity [7], related to increased cardiovascular morbidity and mortality [8,9]. Physical inactivity accelerates these impairments in cardiac autonomic function and contributes to increased cardiovascular disease risk [10,11,12].

Regular physical activity is important at any age to reduce the risk of cardiovascular diseases and prevent age-related decline of cardiovascular health. Aerobic training is recommended for reducing cardiovascular disease risk and all-cause mortality [13]. Furthermore, resistance training is recommended for increasing muscular strength, bone mass, improving quality of life, and reducing cardiovascular disease risk as well [14]. To improve all substantial aspects of cardiovascular health, combining resistance and aerobic training is recommended [15]. This is especially true for an aging population when daily physical activity and physiological functions decrease. However, less than 40% of middle-aged and older adults meet aerobic exercise guidelines, due in part to time availability-related barriers [16].

Heart rate variability (HRV) reflects the fluctuations between consecutive heart beats and non-invasively evaluates the balance of the autonomic nervous system (ANS) [17,18]. These fluctuations are the result of the complex interaction between the sympathetic and parasympathetic nervous system [19]. Increased variability reflects a good health condition and adaptability to internal and external stimuli [17,20,21]. Relatively reduced HRV is an independent predictor of cardiovascular disease, including sudden cardiac death and mortality in middle-aged and older adults [22,23,24,25,26,27]. Moreover, HRV is an established tool in the diagnosis of cardiac autonomic neuropathy in diabetes mellitus [21,28]. Thus, HRV indicates cardiac autonomic control and is a clinically useful tool for detecting disturbances in the cardiovascular and ANS, and is an important marker of adverse prognosis [21,28].

HRV can be described with time-domain, frequency-domain, and non-linear measures. A comprehensive overview of the different measures and their physiological backgrounds can be found in the referenced literature [21,29,30].

A depressed HRV in older individuals compared to younger individuals was found in a number of cross-sectional studies [31,32,33,34,35,36,37]. The exact mechanisms behind this age-related decline in HRV are not precisely known. However, the main reason may be the reduced parasympathetic modulation of cardiac autonomic regulation [38]. Other factors such as lifestyle, medication, and diseases of the cardiovascular system may also play a role and impede the significance of longitudinal studies [39,40].

The beneficial effects of exercise on cardiac autonomic control in young [41] and older adults [42,43] and in patients with certain diseases [44,45,46] has already been demonstrated in recent reviews. However, there is still no comprehensive literature review on the effects of different exercise interventions on cardiac autonomic control in healthy middle-aged adults. The literature shows that this age group is often neglected when planning physical interventions, although building and maintaining physical fitness and cardiovascular health in the midlife period is essential for health in later years. Particularly, it is unclear which kind of exercise modality is appropriate and how the training load should be designed to improve cardiac autonomic control and reduce cardiovascular risk in middle-aged adults. The aim of this review is to fill this gap. We will distinguish between four types of physical interventions representing the main physical training modalities: endurance (aerobic), resistance, coordinative, and multimodal training programs. Endurance and resistance training were considered because they represent major types of exercise to improve cardiorespiratory fitness and muscular strength, respectively. Coordinative training interventions were also considered as the effects of these types of interventions on cardiac autonomic control have been previously disregarded. Coordinative training programs are designed to improve sport-specific skills and include acyclic movements. Regarding multimodal interventions, we considered programs consisting of at least two types of exercise modalities (e.g., endurance and resistance). Multimodal interventions are becoming more popular because they allow for the simultaneous improvement of multiple conditioning skills and provide variety during the training process as opposed to interventions that only improve single skills (e.g., aerobic endurance). Resting state HRV is regarded as an appropriate variable to capture cardiac parasympathetic activity [47] and monitor training status [48] but it represents only one aspect of cardiovascular and autonomic health. Therefore, other cardiovascular health factors, assessed in the retrieved interventions, were extracted and compared with the results of HRV. This approach was adopted to obtain a more sophisticated view on the improvement of cardiovascular health. The following health factors were extracted from the retrieved interventions: body fat (BF), body mass (BM), body mass index (BMI), blood pressure (BP), baroreflex sensitivity (BR), heart rate recovery (HRR), VO_2_ max or VO_2_ peak, and waist-to-hip ratio (WR). The whole procedure of the current review follows a previous review of the same authors considering young adults [41]. As a preservation of the adaptability of the cardiovascular system in older adults is assumed [49] and regular physical activity is positively associated with autonomic function [50], we hypothesized beneficial effects of physical interventions on HRV and secondary health factors in healthy middle-aged adults.

## 2. Materials and Methods

This systematic review followed the Preferred Reporting Items for Systematic Reviews and Meta-Analysis (PRISMA) statement for reporting systematic reviews [51]. A qualitative summary on the effects of exercise interventions on cardiac autonomic control and selected cardiovascular health factors in middle-aged adults were provided. Cardiac autonomic control was indexed as resting HRV. In addition, the methodological quality of the studies using two quality assessment tools (TESTEX and STARD_HRV_) were summarized.

### 2.1. Data Sources and Search Strategy

The electronic databases PubMed, Scopus (Elsevir), SPORTDiscus, Ovid, and Cochrane Library were searched for studies from 1 January 2005 to 8 September 2020 using the following terms: (resistance training OR resistance exercise OR strength training OR strength exercise OR aerobic training OR aerobic exercise OR physical training OR physical exercise OR multimodal training OR multimodal exercise OR coordinative training OR coordinative exercise) AND (heart rate variability OR HRV OR cardiac autonomic control OR autonomic function OR parasympathetic activity OR parasympathetic nervous system OR cardiac vagal tone OR autonomic cardiac modulation OR vagus nerve OR vagal tone OR vagal activity).

### 2.2. Inclusion and Exclusion Criteria

The inclusion criteria for relevant studies were: (1) involving at least ten healthy participants aged between 45 and 60 years old on average without diseases relevant for HRV analysis in the training group (please see for a detailed description of diseases relevant for HRV analysis [40]); (2) with physical training intervention with a minimum of four weeks and eight training sessions; (3) randomized controlled trials, quasi-experimental trials, crossover controlled trials, or controlled trials without randomization; (4) with the measurement of at least one HRV parameter at the resting position before (pre) and after (post) the intervention through a Holter ECG or chest belt; (5) studies with a 24 h ECG measurement when a short-term recording segment at the resting position was analyzed; (6) full-text in the English or German language; and (7) with human participants. Exclusion criteria were: (1) studies with participants with a diagnosis of dementia, mental diseases, neurological diseases, endocrinological diseases (diabetes and thyroid gland disease), cardiac diseases, hypertension, Parkinson’s disease, or other health-related diseases; (2) measuring acute exercise effects or HRV during exercise; (3) single-case studies, review articles, short communications, letters with insufficient information to analyze the results, guidelines, theses, dissertations, qualitative studies, scientific conference abstracts, or studies on animals; (4) 24 h ECG recording without short-term analysis at the resting position; (5) HRV assessment through recording the pulse rate manually or through photoplethysmography; and (6) studies with professional athletes.

### 2.3. Data Collection and Analysis

#### 2.3.1. Selection of Studies

Retrieved articles were transferred to the Citavi 6 reference manager (Swiss Academic Software, Wädenswil, Switzerland) and duplicates were removed. The first screening of the articles was made by analyzing titles and abstracts. Subsequently, two researchers (B.G. and B.T.) independently analyzed the full-texts of each relevant article based on the criteria for inclusion and exclusion. The references of the eligible articles were screened to retrieve further articles. Inconsistent decisions were solved by discussion.

#### 2.3.2. Data Extraction

Based on the PICOS approach [52], the following data were extracted from the selected articles: sample characteristics (sample size, age, and gender), HRV protocol (method (ECG or chest belt), respiration (paced or spontaneous), position (supine, sitting, and standing), sampling frequency), HRV parameters, secondary health factors (BF, BM, BMI, BP, BR, HRR, VO2 max, and WR), recording length, characteristics of the intervention (type, duration of intervention, and sessions per week), and control group. Additionally, resting heart rate (RHR) and mean RR interval (mRR) were also extracted.

#### 2.3.3. Quality Assessment

The methodological quality of the studies was assessed using the “Tool for the Assessment of Study Quality and reporting in Exercise (TESTEX) scale” [53]. The TESTEX scale consists of twelve criteria with a maximum score of 15 points. The methodological quality of HRV recording, processing, and analyzing was assessed using the tool STARD_HRV_ [54]. It includes 25 criteria with a maximum score of 25 points. Two authors (B.G. and B.T.) evaluated the studies’ quality independently. Based on our research questions and the included studies, slightly modified versions of the TESTEX and STARD_HRV_ were implemented (please see Supplementary Material Tables S1 and S2). Disagreement was solved by discussion.

#### 2.3.4. Data Synthesis and Analysis

The characteristics of the selected studies are described in Table 1. Table 2 displays the pre–post change of heart rate-related parameters and secondary health factors, as well as the scores in STARD_HRV_ and TESTEX. Positive, negative, or no changes between pre and post intervention were collected for all heart rate-related parameters and secondary health factors. Increases were marked with an upward arrow, decreases with a downward arrow, and no changes with a horizontal arrow. Significant changes based on the criteria of the studies’ authors were marked with an asterisk (Table 2) and delta values (post minus pre) were calculated.

## 3. Results

### 3.1. Study Selection

**Figure 1:**
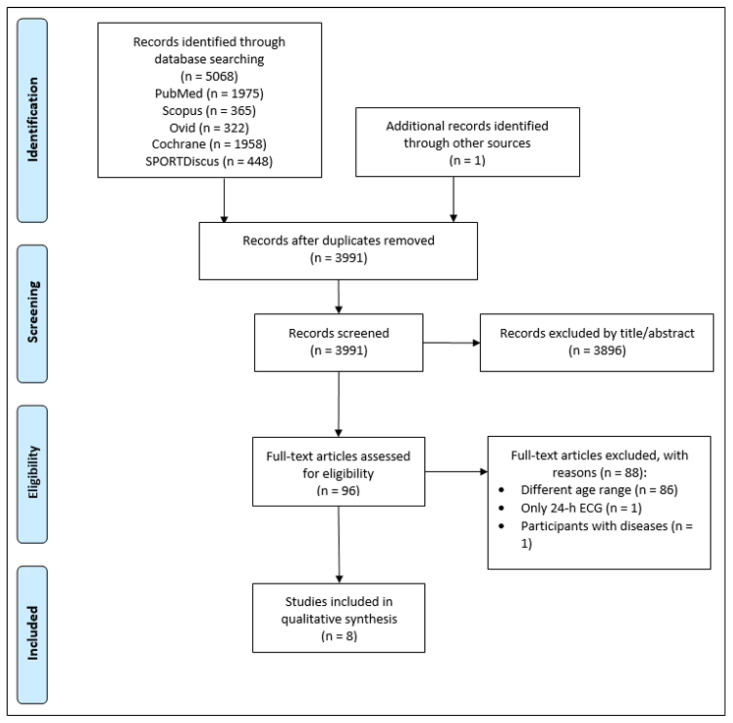
PRISMA flow diagram showing identified, included, and excluded studies.

The systematic search identified 5068 records. In total, 1078 duplicates were removed and one study was identified through the references of the final sample. Finally, 3991 titles and abstracts were screened. Of these, 3896 articles were initially excluded. In total, 96 full-text articles were further assessed for eligibility. Finally, eight articles met our inclusion criteria and were considered for the qualitative analysis. The PRISMA 2009 flow diagram illustrates the selection process (Figure 1).

### 3.2. Study Characteristics

The characteristics of the selected studies are described in Table 1. More details of the studies are available in the supplementary material (additional Table S3).

The eight studies comprised a total of 381 participants (264 women; 117 men). In total, 306 individuals were allocated to the training groups and 75 individuals to the control groups. Four studies included a control group [3,55,56,57] and one study assessed the effects of two training interventions using a crossover design with a washout period between both intervention periods [58]. The total sample sizes ranged from 12 [59] to 93 participants [55]. The mean age ranged from 47.3 [60] to 59 years [58,59,61]. The mean age of the training groups was 55.36 years and of the control groups 55.89 years. In only two studies, both genders, men and women, participated [58,59]. In five studies exclusively women [3,56,57,60,61] and in one study exclusively men participated [55]. All participants were in a generally healthy condition with no severe diseases affecting cardiac autonomic control. Participants with a systolic blood pressure above 120 mmHg or a diastolic blood pressure above 80 mmHg participated in one study [58] but these participants had no physical limitations or took any blood pressure-regulating medication. However, it is still possible that hypertensive participants with a blood pressure above 140/90 mmHg exercised. Overweight participants with an average BMI of 26.2 kg/m^2^ took part in the study of Jakubec et al. [60]. All studies focused on sedentary participants without practicing regular physical activity.

Endurance intervention was the most applied exercise intervention [3,48,51,52]. One study [51] assessed the effects of two training intensities in a crossover study: endurance training with low (33% of heart rate reserve) and high (66% of heart rate reserve) intensity. Between both intervention periods, a ten-week washout period was inserted. Resistance training interventions were implemented in three trials [3,48,54]. Two trails used multimodal interventions [3,50] and two investigations used coordinative training [49,53]. The last two studies implemented aerobic dance or step dance [53] and step aerobics [49]. Two studies assessed the effects of endurance and resistance training isolated and the effects of a combination of endurance and resistance training in the multimodal training group [3,48]. The average intervention duration was 17.25 weeks, lasting from 10 [49,51] to 24 weeks [52,53]. All interventions applied two to four sessions per week.

Cardiac activity was recorded via an ECG in four studies [56,58,59,61]. A chest belt was used in the other investigations [3,55,57,60]. Only three authors reported the sampling frequency of their deployed device [55,56,61]. A spontaneous breathing protocol was used in four studies [3,55,57,58]. Participants were requested to breath 15 times per minute in one study [59] and 12 times per minute in another study [61]. Two studies failed to report their breathing protocol [56,60]. ECG was recorded in the supine position in four studies [3,55,57,60] and in the sitting position in one study [58]. The other three studies did not report the recording position [56,59,61]. Five studies recorded ECG for five minutes [55,56,59,60,61]. Three studies analyzed a certain amount of heart beats but recorded for at least five minutes: 512 [58], 600 [3], and 1000 heart beats [57].

Frequency-domain parameters were assessed in all studies except in one [57]. Four studies analyzed time-domain parameters [3,56,57,60] and four studies analyzed non-linear parameters [3,55,57,61]. The two most commonly used HRV indices were LF and HF (ms^2^, nu, or ln). Six studies analyzed them [3,55,56,58,60,61]. Additionally, respiratory sinus arrythmia (RSA) with a frequency range between 0.2 and 0.3 Hz was used in one study [59]. Although SDNN and RMSSD are very common HRV parameters, they were analyzed only in two [3,56] and one trial [56], respectively. The very unusual parameter of mean squared successive differences (MSSD) was analyzed in one study [60]. α1 was the most commonly used non-linear parameter [55,57]. RHR (resting heart rate) was measured in all studies except one [57].

Two investigations did not report any secondary health factor [55,57]. VO_2_ max was recorded in four studies [55,56,59,60]. Three studies investigated resting BP [58,59,61]. Three studies measured BM and BMI [56,59,60]. BF [56], BR, HRR [58], and WR [59] were measured in only one study each.

### 3.3. Heart Rate-Related Variables

#### 3.3.1. Endurance Training

Four studies using endurance training interventions were retrieved from the literature search [3,55,58,59]. While two studies implemented aerobic training on a bicycle [3,55], the other two studies used a mix of walking, jogging, running, cycling, and stepping [58] or aerobic training on a treadmill, elliptical trainer, or bicycle [59]. Two interventions lasted for 21 weeks [3,55], one study for ten weeks [58], and the last study for 24 weeks. In two studies, participants had to exercise two times per week [3,55], in one study three times per week [58], and in one study even four times per week [59]. However, in the latter study, one training session was not supervised. While two studies examined women and men [58,59], one study included only women [3] and one study only men [55]. RHR decreased in three studies [3,58,59]. RHR decreased by 4.1 bpm and mRR increased by 124.7 ms after the intervention of Deley et al. [59]. RHR decreased by 4.0 bpm after the intervention of Karavirta et al. [55]. In the study of Cornelissen et al., RHR decreased even after low (∆ −6.1 bpm) and high (∆ −5.5 bpm) intensities [58]. The same study reported a significant increase of TP (total power) after the low (∆ 0.37 ln ms^2^) but not after the high intensity training. RSA (respiratory sinus arrhythmia) significantly increased (∆ 142.9 ms^2^) after one study [59]. No changes in cardiac autonomic control were detected in two studies [3,55].

#### 3.3.2. Resistance Training

Resistance training interventions were implemented in three studies [3,55,61]. While the number of repetitions was constant throughout the intervention period in one study [61], intensity increased and the number of repetitions decreased in the other two studies [3,55]. All three interventions implemented two training sessions per week. However, one study lasted for twelve weeks [61] and the other studies for 21 weeks [3,55]. Heart rate-related variables did not change significantly after any of the three interventions.

#### 3.3.3. Coordinative Training

Forms of coordinative training interventions were implemented in two studies [56,60]. In one study [56], participants performed step aerobics for ten weeks. In the other study [60], participants performed aerobic dance or step dance for 24 weeks. In both interventions, participants exercised three times per week. While no changes were detected in one study [60], the other study [56] reported significant increases in HF nu (∆ 6.83) but significant reductions in SDNN (∆ −7.33 ms), CV (∆ −0.81), NN50 (∆ −5.81), pNN50 (∆ −0.02 %), LF nu (∆ −6.83), and LF/HF (∆ −0.79).

#### 3.3.4. Multimodal Training

The literature review retrieved three multimodal interventions [3,55,57]. A combination of functional training and walking was implemented in one intervention [57]. Participants of this study exercised three times per week for 16 weeks. Four training sessions per week for 21 weeks with two endurance sessions on a bicycle and two whole-body resistance training sessions per week were implemented in the other two interventions [3,55]. RHR significantly decreased after one intervention (∆ 1.0 bpm) [55]. α1 significantly improved after the interventions of Karavirta et al. [55] (∆ −0.07) and Rezende Barbosa et al. [57] (∆ 0.073). Interestingly, it decreased in one study [55] but increased in the other study [57]. However, in both studies, α1 approached the optimal value of 1. Finally, α1/α2* (∆ 0.13) significantly improved in the latter study. There was also a significant increase in SD1 (∆ 3.6 ms) in the training group compared to the control group [57].

### 3.4. Secondary Health Factors

#### 3.4.1. Endurance Training

Three of four endurance interventions assessed secondary health factors [55,58,59]. VO_2_ max improved after the interventions of Deley et al. by 3.1 mL/min/kg [59] and Karavirta et al. by 11.9% [55]. SBP (systolic blood pressure) at the resting state decreased after the intervention of Cornelissen et al. by 3.8 mmHg in the training group exercising with low intensity and by 6.0 mmHg in the group exercising with high intensity [58]. A significant reduction was also observed during a physical exercise test at 40 Watt in the low (∆ −2.4 mmHg) and high intensity group (∆ −2.7 mmHg). SBP at the resting state also decreased after the intervention of Deley et al. (∆ −7.2 mmHg) [59]. HRR [58] improved in one study but no values were given. Measures of body composition (∆ BF: −1.9%; ∆ BM: −2.7kg; ∆ BMI: −0.9 kg/m^2^) as well as BR (∆ 26%) improved after the aerobic intervention of Deley et al. [59].

#### 3.4.2. Resistance Training

Two of three resistance interventions assessed secondary health factors [55,61]. Both interventions did not detect significant changes in any of the assessed cardiovascular health factors.

#### 3.4.3. Coordinative Training

VO_2_ max improved after both coordinative training interventions. It significantly improved by 3.67 mL/kg/min after the intervention of Jakubec et al. [60] and by 4.91 mL/kg/min after the intervention of Shen and Wen [56]. Furthermore, BM (∆ −1.19 kg) and BMI (∆ −0.37 kg/m^2^) significantly decreased after the aerobic dance intervention [60].

#### 3.4.4. Multimodal Training

One of the three multimodal training interventions assessed one secondary health factor, namely VO_2_ max. In this study, VO_2_ max significantly improved after the intervention by 10.1% [55].

### 3.5. Quality Assessment

The total scores of TESTEX and STARD_HRV_ are shown in Table 2. The possible maximum scores are 15 points for TESTEX and 25 points for STARD_HRV_. The score of the TESTEX ranged between 4.5 [60] and 9.0 [3,56]. On average, the studies scored 7.00 points. However, three studies did not include a control group [59,60,61] and one study used a crossover design without a passive control group [58]. These studies could not achieve more than eight points. All studies specified the eligibility criteria and, in case of a control group, the randomization process, and showed no significant differences between the training and control group in any of the assessed variables at baseline. A number of studies failed to provide any information regarding blinding of assessors (4 of 4 studies), the intention-to-treat analysis (7/8), and the activity monitoring in the control groups (4/4). All scores for each item are displayed in Table S4. 

The average score of the STARD_HRV_ was 18.69 and ranged between 16 [60] to 20.5 points [3,55,61]. All studies fulfilled the items 1, 2, 3, 5, 7, 14, 20, and 24. Nearly all studies provided enough information about the pre-testing conditions, setup of the used device, interbeat artefact identification method, metrics used, and specification of the frequency bands. Contrarily, only two studies stated how many participants had to be excluded due to a high amount of artefacts [3,60]. Additionally, one study [55] stated that participants with more than 15% noise or ectopic beats were ruled out from HRV analysis but did not state how many participants were excluded. Only one study reported how the intended sample size was calculated [57]. Three studies provided information about a stabilization period prior to the recording [55,60,61]. Finally, no study provided full information about the artefact cleaning method and percentage of beats corrected. See Table S5 for detailed results.

## 4. Discussion

### 4.1. Purpose and Main Findings

This systematic review was conducted to summarize the existing literature on the effects of different physical training modalities on cardiac autonomic control and secondary health factors in healthy middle-aged adults. HRV was used as parameter of cardiac autonomic control. As the aging process affects cardiac autonomic control differently [40], we performed an age-differentiated analysis of the study results. In this review, we restricted the results to middle-aged adults up to the age of 60 years, as postmenopausal hormonal changes are not completed until the age of 60 [62,63]. The literature search retrieved eight studies fulfilling all inclusion criteria. The majority of the selected studies demonstrated beneficial effects of exercise training interventions on cardiac autonomic control and secondary health factors. Two of four endurance interventions reported significant improvements in cardiac autonomic control [58,59]. Small improvements were detected in one study [55] and one study did not show clear results [3]. Regarding resistance training interventions, the literature search retrieved no significant changes in HRV. Small improvements [3], small impairments [61], and inconsistent changes [55] were reported. The two coordinative training studies [56,60] yielded inconsistent results, though stronger improvements were reported for participants with lower aerobic capacity and lower HRV values at baseline [60]. Finally, two multimodal exercise interventions demonstrated significant improvements [55,57] but one study could not detect any improvement [3]. The most pronounced effects were found for frequency-domain parameters. Significant improvements in secondary health factors after exercise interventions were reported in five studies [55,56,58,59,60]. One study detected no significant effects on health parameters [61]. The results of both quality assessment scales, TESTEX and STARD_HRV_, revealed deficits in the description of methods and study-reporting in some trials.

### 4.2. Heart Rate-Related Variables

#### 4.2.1. Endurance Training

Four studies implemented endurance training interventions. While RHR was significantly reduced after three interventions [55,58,59], significant improvements in cardiac autonomic control were detected in two studies: TP [58] and RSA [59]. A shortcoming of the study of Deley et al. [59] was that only one HRV parameter was considered, namely RSA. Interestingly, there was no difference between low and high intensity training in the crossover study of Cornelissen et al. [58], though significant improvements were found only after the low intensity training. This finding contradicts study results favoring higher intensities for improving cardiac autonomic control [64]. Moreover, the improvements were found only in the parameter TP but not in LF nor HF [58]. One explanation for the lack of significant improvements could be the relatively short intervention period of ten weeks. Furthermore, as stated by the authors, spontaneous respiration was one limitation of this study as the intervention could have changed the respiratory rate during ECG recording. However, significant effects on respiratory rate after exercise training with moderate intensity was not observed in another study [65]. Both training interventions of Karavirta et al. [3,55] did not detect significant changes in HRV despite the relatively long intervention period of 21 weeks. However, the low intensity may account for the non-significant changes in resting HRV [3].

In summary, the results indicate beneficial effects on cardiac autonomic control after endurance training interventions in middle-aged adults. Additionally, endurance training seems to be appropriate to reduce RHR. The current findings are in line with previous reviews showing beneficial effects of endurance training on cardiac autonomic control [41,42]. The results suggest that at least three training sessions per week are necessary to induce HRV changes. Additionally, even longer intervention periods do not lead to HRV changes when the training dose is too low [66,67,68]. Alongside exercise duration, intensity, and frequency [59], baseline level, setting (supervised, group, or home training), and genetic factors should be considered when planning training programs for middle-aged adults. The type of exercise also plays an important role as participants of an interventional study have different preferences. Additionally, the results of the four studies show that the application of different types of exercise leads to better results [58,59] than training with only one type of exercise [3,55]. Finally, we suggest to individualize the training load as a proper approach to enhance cardiac autonomic control.

#### 4.2.2. Resistance Training

Three resistance training interventions showed no significant effects on cardiac autonomic control or RHR [3,55,61]. The authors of [61] mentioned the lacking control for the effect of the menstrual cycle on autonomic modulation as one limitation. However, the influence of the menstrual cycle on cardiac autonomic control is discussed controversially [61]. Furthermore, the short intervention period of 12 weeks and lack of variation in the training components (i.e., intensity and number of repetitions and sets) might have been insufficient to induce adaptations in the cardiac autonomic control [61]. Contrarily to the study of Gerhart et al. [61], both studies of Karavirta et al. [3,55] used only chest belts to measure heart rate. Another limitation of all studies was the small number of HRV parameters considered for analysis. Two studies did not report time-domain parameters [55,61] and one study only reported SDNN as time-domain parameter, CI as non-linear measure, and HF, LF, and LF/HF as frequency-domain parameters [3].

In summary, resistance training interventions seem to be inappropriate to stimulate cardiac autonomic control in healthy middle-aged adults. There were no changes obtained with a periodized intervention [3,55], nor with a constant intensity throughout the intervention [61]. This finding contradicts previous reviews showing beneficial effects in young adults [41,69]. Two weekly training sessions are probably not enough to elicit changes in autonomic modulation. Interestingly, isometric resistance training has been shown to improve cardiac autonomic control [70]. The absence of adaptability of the cardiac autonomic system might also be the result of the old age of the participants as an age-related increase of vascular stiffness induces negative effects on blood flow [71]. However, given the small number of studies, more research is necessary to elucidate the effects of resistance training in middle-aged adults. 

#### 4.2.3. Coordinative Training

The literature search retrieved two studies applying coordinative training programs in women but none for men: aerobic dance or step-dance [60] and step aerobics [56]. The results are inconclusive as one study [60] failed to show significant changes, while significant improvements of HF nu and significant decreases in some other indices (SDNN, NN50, NN20, LF nu, and LF/HF) were found in the other study [56]. The training volume differed between the interventions as one intervention [56] lasted for ten weeks, while the other intervention [60] lasted for 24 weeks, but both interventions implemented three sessions per week. However, training volume could not explain the diverging results. Contrarily, exercise intensity might be one explanation as one intervention reported a stronger shift towards parasympathetic activity in women who exercised at higher intensities or had higher adherence to the intervention [60]. Furthermore, the initial HRV level is another decisive factor mediating the improvements in cardiac autonomic control [72,73]. Women with lower HRV at baseline tend to improve cardiac autonomic control more than women with higher HRV values at baseline or with higher aerobic capacity. The ECG measurement protocol might be another explanation for the inconclusive results as some shortcomings were detected. While Shen and Wen used an ECG device, they failed to report respiration protocol and participants’ position during the recording [56]. An ECG chest belt was used in the study of Jakubec et al. [60] but the respiration protocol, recording frequency, and daytime of the measurement were not reported. 

In conclusion, aerobic dance demanding high coordinative skills could be an effective activity for people with low aerobic capacity but not for people with an already good level of cardiac autonomic control or a low level of coordinative skills [60]. Finally, assessing changes in cardiac autonomic control with coordinative training demands accurate control of the training load. Therefore, we suggest to control and monitor the training load during sessions by measuring the heart rate [74], which has not been done in these studies [56,60].

#### 4.2.4. Multimodal Training

Multimodal training interventions were applied in three studies using resistance and endurance training [3,55,57]. While two studies reported significant improvements [55,57], no significant changes were found in the third investigation [3]. Interestingly, although the studies [3] and [55] applied the same training protocol, they revealed diverging results. Both studies implemented the same endurance and resistance training, and participants exercised four times per week (two resistance and two endurance training sessions). One explanation could be that men participated in one study [55] and women in the other one [3]. However, Karavirta et al. [55] reported improvements in α1, which was not assessed in the other study [3]. All three studies used chest belts for recording heart rate while participants were lying in the supine position and breathed spontaneously. However, recording length and analyzed HRV parameters differed. One study recorded for five minutes [55], one study analyzed approximately 600 heart beats [3], and one study 1000 heart beats [57]. RHR and frequency-domain parameters were not assessed in the investigation of Rezende et al. [57] and no time-domain parameters were assessed in the study of Karavirta et al. [55]. 

Another interesting finding was that the combination of endurance and resistance training improved heart rate dynamics more compared to endurance training alone [55]. It has to be clarified whether this synergistic effect is due to the greater training volume (four vs. two sessions per week) or due to other mechanisms of training adaptation. Whether the heterogeneity of responses increases when combining endurance and resistance training may depend on genomic factors [3]. These inconclusive results regarding the heterogenous effects of combined and isolated training support the need for the individualization of training prescription based on the health state and fitness aims of the participants [3]. The authors of [57] suggest functional training as a well-established exercise modality to prevent changes in cardiac autonomic control induced by menopause because it follows everyday gestures and activities, and are widely used in clinical practice and for rehabilitation. 

In summary, multimodal training interventions showed a tendency of improved cardiac autonomic control but there is a need for further investigations. We suggest to assess linear as well as non-linear HRV parameters and compare the effects of multimodal with isolated training interventions to differentiate the effects of different exercise modalities on cardiac autonomic control.

### 4.3. Secondary Health Factors

#### 4.3.1. Endurance Training

Aerobic exercise training is a recommended training modality especially for middle-aged and older adults to improve cardiovascular function [16]. The current findings revealed significant improvements in cardiovascular health in three studies [55,58,59]. As expected, endurance training positively affected VO_2_ max in two studies [55,59]. Interestingly, accelerated HRR was significantly improved after low and high intensity training but was more pronounced after the high intensity training [58]. Additionally, both exercise groups equally decreased SBP. Moreover, the significant reduction of BP was not only found at rest but also during a submaximal physical exercise. This result is of significance as the SBP of participants before the intervention was ≥ 120 mmHg. The positive influence of endurance training on BP has been confirmed by another study [59], also showing a beneficial influence on body composition. These results support the positive effects of endurance training on autonomic control, indicated by an increase in RSA and decrease in RHR. Overall, the results indicate an improved health status of the participants after the intervention. Despite the general known positive effects of endurance training on cardiovascular health, future studies should more thoroughly investigate the effects of different types of endurance training interventions with different intensities and volumes on secondary health factors. The current findings indicate a positive relation between cardiac autonomic control and secondary health factors.

#### 4.3.2. Resistance Training

The results regarding the effects of resistance training interventions on secondary health factors is limited as only two studies assessed the effects on BP [61] and VO_2_ max [55]. Both interventions did not significantly lead to positive changes. Due to relatively low BP values at baseline (119/80 mmHg) in the intervention of Gerhart et al. [61], the absence of BP changes was not surprising. These results are in line with another study applying resistance training in young adults [75] but contradicts studies using isometric resistance training [70,76]. In conclusion, the effects on dynamic resistance training in middle-aged adults has to be investigated with (pre)hypertensive participants. 

The absence of changes in VO_2_ max after the intervention of Karavirta et al. [55] might be the result of the low training volume (two sessions per week) and probably the low effect of the resistance training on the cardiovascular system. This finding supports the absence of changes in cardiac autonomic control. The result contradicts one study with younger participants [77] but are in line with another investigation [78]. However, resistance training has been proven to be an adequate exercise modality for older adults to preserve independence in daily life due to the increase in muscular strength [79].

#### 4.3.3. Coordinative Training

Previous studies support the positive effects of dance training on aerobic capacity, showing similar effects on the aerobic power involved in, for example, running [80], walking, and jogging [81]. Therefore, (aerobic) dancing could be a suitable exercise modality especially for middle-aged adults with low aerobic power and/or limited experience in physical training because it combines psychological as well as physiological aspects [60]. However, the effects on body composition are controversial as Jakubec et al. [60] reported significant reductions in BM and BMI, but Shen and Wen [56] did not found changes in BF, BM, or BMI. One possible explanation might be the short intervention period in the latter study that was not sufficient to elicit changes in body composition. Furthermore, body composition at baseline should also be considered as the BMI of the participants in Jakubec et al. [60] was higher compared to the BMI of the participants in Shen and Wen [56]. Interestingly, the results of the secondary outcomes do not support the results of the HRV analysis, where no clear changes were detected. Therefore, we suppose that effects on cardiac autonomic control require a longer intervention period than changes in aerobic capacity or body composition. 

Dancing and other group-based training programs such as team sports also incorporate social aspects which are a motivating factor [82]. For the future, we recommend to include men as well to investigate the effects on both genders. Furthermore, we suggest to monitor the training load to better assess the effects on cardiovascular health.

#### 4.3.4. Multimodal Training

Only one study assessed secondary health factors [55] and reported positive changes in VO_2_ max. Interestingly, the authors did not detect any difference in the change of VO_2_ max between the multimodal and endurance training group, despite the fact that the endurance training group exercised two times per week and the multimodal training group four times per week. Therefore, the additional resistance training had no disturbing effect on the improvement of aerobic capacity. This improvement of VO_2_ max might be the result of the high training volume consisting of four sessions per week for 21 weeks and supports the positive change in cardiac autonomic control. However, four sessions per week seems to be the upper limit for untrained middle-aged individuals [3]. In conclusion, more research is necessary to elucidate the effects of combined exercise training interventions in middle-aged adults. An important issue concerns whether the effects on cardiovascular health are confounded by the application of different exercise modalities and how baseline state confounds the effects.

### 4.4. Possible Mechanisms behind Autonomic and Cardiovascular Adaptations

Several physiological mechanisms may be responsible for the adaptations in the autonomic and cardiovascular system. Decreased sympathetic outflow and decreased intrinsic heart rate have been suggested to reduce resting heart rate and increase vagally-mediated HRV measures [83]. Furthermore, decreased sympathetic outflow in cardiovascular regions of the brain stem and increased cardiac vagal tone after physical exercise could mediate a faster heart rate recovery [84], indicating a better cardiovascular state [85]. Stimulation of nitric oxide syntheses or the suppression of angiotensin II may also contribute to enhanced vagal activity [86,87]. Some studies also suggest that the increase of carotid elasticity, the reduced vascular resistance, and the decrease in the stiffness of barosensory vessels positively affects baroreflex sensitivity and blood pressure [4,59,88]. However, the exact mechanisms and the optimal dose–response relationship of the different exercise modalities have to be elucidated in further studies.

### 4.5. Quality Assessment

#### 4.5.1. TESTEX

The methodological and study-reporting quality was assessed via the TESTEX scale [53]. The scores ranged from 4.5 [60] to 9.0 points [3,56] and the average score was 7.00. However, four studies did not use a control group and therefore could not achieve more than 8 points. The average score was 7.88 points when excluding these studies and this is considerably lower than the score in our previous review investigating the effects of different exercise modalities in young adults [41]. In that review, the average score was 7.90 for all studies and 8.93 when considering only trials with control groups. This relatively low score was mainly the result of four factors that were fulfilled by none of the studies: allocation concealment, blinding of assessors, intention-to-treat analysis, and activity monitoring in the control groups. Therefore, these aspects should be considered in future studies to ensure a good study quality.

#### 4.5.2. STARD_HRV_

The methodological quality of HRV recording, processing, and analyzing was assessed with the tool STARD_HRV_ [54]. The average score of the studies was 18.69 and ranged from 16 [60] to 20.5 points [55]. This is a minimally better score than in our previous review with young adults [41]. All studies fulfilled the first three criteria (identification as a study of validation; structured summary; and scientific and practical background). A within-subject design, statement of eligibility criteria, description of measures, baseline demographics of participants, and full study protocol were also provided in all studies. However, the intended sample size was calculated only in one study [57]. Additionally, we also detected shortcomings in two other criteria (reasons for missing data, alongside percentage missing and how it was handled; and artefact cleaning methods and percentage of beats corrected). A further aspect is the recording length as it has an influence on the magnitude of the HRV values. Therefore, it is recommended to standardize the recording length for short-term measurements [89]. Five minutes after an adequate stabilization period is supposed to be an optimal length. Future investigations should thoroughly adhere the criteria of both TESTEX and STARD_HRV_, enabling a better comparability between study results and repeatability of the existing investigations.

### 4.6. Strengths and Limitations

This review has a few limitations. First, only healthy middle-aged adults were considered. Thus, the results are not generalizable to diseased or older adults. Second, non-randomized controlled trials without control groups were included in this review as well because we intended to give a comprehensive overview of the existing literature. Third, we desisted from performing a meta-analysis and instead conducted a qualitative analysis. A meta-analysis would provide the opportunity to quantify the effects of the different exercise modalities on cardiac autonomic control. However, the small number of studies in each exercise modality, the heterogeneity of the studies regarding the gender-distribution, and the methods of HRV analysis hampered us from performing a quantitative analysis. Furthermore, only two studies examined women and men, and only one study was conducted exclusively with male participants. Due to this imbalance of gender-distribution, no clear statement regarding the effects on HRV in men and regarding the differences between men and women could be made. Finally, only original articles in the English and German language (no article in German was in the final sample) were included in the literature search.

Our systematic review has some strengths. It is the first review focusing on the effects of different physical interventions on cardiac autonomic control in healthy middle-aged adults. In addition, we also looked for different secondary health factors, which are important especially for middle-aged adults for the prevention of cardiovascular diseases. Finally, this is the first review evaluating the methodological and study-reporting quality with a special focus on HRV recording, processing, and analyzing. The results of the quality assessment tools demonstrated some shortcomings in the methodological quality of the studies. Therefore, we would like to encourage investigators to follow the criteria of TESTEX and STARD_HRV_.

### 4.7. Practical Implications 

The main finding of this systematic review was that two endurance training interventions [58,59], two combined endurance and resistance training interventions [55,57], and one step aerobics intervention [56] lead to significant improvements in cardiac autonomic control. As the majority of the studies analyzed frequency-domain parameters, the most pronounced effects were found in this domain. However, we strongly recommend to consider also RMSSD as this parameter reflects vagally mediated changes in HRV [19]. Additionally, non-linear parameters should be considered as well, as these indices reflect complex and non-linear cardiovascular regulation [90]. Improvements in secondary health factors were recognized after endurance [55,58,59], multimodal [55], and coordinative training interventions [56,60]. Regular physical activity decreases RHR and BP [58,59], improves HRR [58], prevents age-related declines in BR [59] by decreasing vascular stiffness [4,91] and improving cardiac vagal neural control [59], and improves aerobic capacity [55,56]. However, the small number of studies and participants prevents stronger conclusions regarding the positive effects of different types of exercise on cardiac autonomic control and health factors.

The results are inconclusive regarding which HRV parameters should be considered when evaluating the functioning of the ANS. Therefore, we recommend to analyze linear and non-linear HRV measures to capture also the non-linear interaction of the mechanisms involved in cardiovascular regulation [57]. Furthermore, due to the small number of investigations with male participants (*n* = 3), the data is still quite limited to make definite conclusions about the effects on cardiac autonomic control in men. Therefore, we strongly recommend to investigate the effects of exercise interventions in middle-aged men. Additionally, the small number of studies in the different exercise modalities do not allow any suggestions about the optimal exercise modality or optimal dose of exercise training, even if endurance training seems to be the most appropriate exercise modality. Therefore, further investigations are necessary to investigate the effects of different training loads (i.e., frequency and intensity). Given that only half of the studies used a control group, future studies should compare the results of the intervention with a passive control group. Furthermore, the menstrual cycle and the menopausal status should be considered when women are included in the study sample as these factors affect cardiac autonomic control and cardiovascular risk [92]. We also suggest to monitor training sessions using activity trackers (i.e., heart rate monitors) to guide training intensities and make conclusions about the training load. Three to four sessions of (aerobic) exercise per week with moderate to vigorous-intensity lasting on average 40 min per session are recommended to reduce cardiovascular disease risk [93].

## 5. Conclusions

The present systematic review summarized the effects of different exercise interventions on cardiac autonomic control, indexed as resting HRV, and secondary health factors in healthy middle-aged adults. The literature search revealed beneficial effects of endurance and multimodal exercise interventions on cardiac autonomic control and secondary health factors. These improvements may be of clinical importance as cardiovascular and cardiac autonomic health decline with age. However, interventions applying resistance training were not able to improve cardiac autonomic control nor secondary health factors. Coordinative exercise interventions showed significant positive effects on secondary health factors but results regarding cardiac autonomic control were inconclusive. Due to the small amount of coordinative and resistance training interventions, no final conclusions about the effects on cardiac autonomic control can be made. 

This systematic review indicates that there is a need to investigate the effects of various physical activities on the autonomic and cardiovascular systems in middle-aged adults. Although the results suggest that endurance training is the best training modality to improve cardiac autonomic control, future studies need to further investigate the effects of different training protocols regarding type, intensity, and volume. Moreover, the autonomic and cardiovascular state before the intervention has to be considered when planning intervention programs. Additionally, as only little research has been done regarding male participants, we suggest to examine the effects on cardiac autonomic control and secondary health factors in both genders. The relatively small number of studies and participants suggests that more interventions should be conducted with middle-aged adults to assess the clinical relevance of different physical exercises to improve cardiac autonomic control and reduce cardiovascular risk. The assessment of the methodological quality of the studies revealed some deficits in the reporting of the methods and HRV processing. Therefore, we strongly encourage to adhere to the methodological standards of recording, processing, and analyzing HRV.

## Figures and Tables

**Table 1:** Characteristics of the studies included.

Study	Participants (Sample Size, Age (Year), and Gender)	HRV Protocol (Method, Respiration, Position, and Sampling Frequency)	HRV Measures	Secondary Outcomes	Analysis Length	Intervention (Type, Duration, and Sessions/Week)	Control Group
[3]	90 (E: 26; R: 26; ER: 21; CG: 17) Age: 49−52 ± 6-8 (E: 52 ± 7; S: 52 ± 8; ES: 49 ± 6; CG: 52 ± 8) 100% women	Polar RS810i belt Spontaneous Supine n. r.	CI, HF, LF, LF/HF, RHR, and SDNN		Approx. 600 beats	Endurance, strength, and combined endurance and strength 21 weeks E and R: 2, ER: 4 sessions	yes
[55]	93 (E: 23; R: 25; ER: 29; CG: 16) Age: 55.6 ± 7.4 100% men	Polar RS810i belt Spontaneous Supine 1.000 Hz	ln HF, ln LF, RHR, and α1	VO_2_ max	5 min	Endurance, strength, and combined endurance and strength 21 weeks E and R: 2, ER: 4 sessions	yes
[56]	44 (TG 22; CG 22) Age: 58.48 ± 0.53 (EG: 57.86 ± 0.64; CG: 59.10 ± 0.83) 100% women	3-lead ECG n. r. n. r. 500 Hz	CV, HF nu, LF nu, LF/HF, mRR, NN20, NN50, pNN20, pNN50, RHR, RMSSD, SDNN, SDSD, TP, and VLF	BF, BMI, BM, and VO_2_ max	5 min	Coordinative (step aerobics) 10 weeks 3 sessions	yes
[57]	39 (TG: 19; CG: 20) Age: TG: 50 ± 4.5; CG: 58.45 ± 4.8 100% women	Polar RS800 belt Spontaneous Supine n.r.	DFA total, RRTri, SD1, SD2, SD1/SD2, TINN, α1, α2, and α1/α2		1000 consecutive beats	Multimodal (functional training and walking) 16 weeks 3 sessions	yes
[58]	36 Age: 59 (range: 55–71) 19 women, 17 men	3-lead ECG Spontaneous Sitting n.r.	HF%, LF%, ln LF/HF, RHR, and TP	HRR, SBP at rest and during ergometer test	512 consecutive beats	Endurance (walking, jogging, running, cycling, and stepping); crossover design: 10 weeks LI or HI + 10 weeks sedentary + 10 weeks HI or LI 10 weeks 3 sessions	crossover design
[59]	12 Age: 59 ± 5 5 women, 7 men	ECG 15 breaths/min n.r. n.r.	mRR, RHR, and RSA	BM, BMI, BR, DBP, SBP, VO_2_ max, and WR	5 min	Endurance (treadmill, elliptical trainer, or bicycle) 24 weeks 4 sessions	no
[60]	44 Age: 47.3 ± 5.4 100% women	Varia Cardio TF4, ECG belt n.r. Supine n.r.	HF, HF%, LF, LF/HF, LF%, MSSD, RHR, TP, VLF, VLF%, VLF/HF, and VLF/LF	BM, BMI, and VO_2_ max	5 min	Coordinative (aerobic dance or step-dance) 24 weeks 3 sessions	no
[61]	23 Age: 59 ± 6 100% women	ECG 12 breaths/min Supine 1.000 Hz	HF nu, LF nu, LF/HF, ln HF, ln LF, ln TP, RHR, and SampEn	BP	5 min	Resistance 12 weeks 2 sessions	no

Abbreviations: BM, body mass; BMI, body mass index; BP, blood pressure; BR, baroreflex sensitivity; CG, control group; CI, complexity index; CV, coefficient of variation; DBP, diastolic blood pressure; DFA, detrended fluctuation analysis; E, endurance training group; ECG, electrocardiography; ER, endurance and resistance training group; HF (nu), power in high frequency range (in normalized units); HF%, relative power in high frequency range; HI, high intensity training; LF (nu), power in low frequency range (in normalized units); HRR, heart rate recovery; LF%, relative power in low frequency range; LI, low intensity training; ln, natural logarithm; mRR, mean RR interval; MSSD, mean squared successive differences; NN20, number of pairs of adjacent NN intervals differing by more than 20 ms in the entire recording; NN50, number of pairs of adjacent NN intervals differing by more than 50 ms in the entire recording; n.r., not reported; pNN20, NN20 count divided by the total number of all NN intervals; pNN50, NN50 count divided by the total number of all NN intervals; R, resistance training group; RHR, resting heart rate; RMSSD, square root of the mean of the sum of the squares of differences between adjacent NN intervals; RRTri, triangular index; RSA, respiratory sinus arrhythmia; SampEn, sample entropy; SBP, systolic blood pressure; SD1, standard deviation of instantaneous beat-to-beat variability extracted from Poincaré Plot; SD2, standard deviation of the long-term variability extracted from Poincaré Plot; SDNN, standard deviation of NN intervals; SDSD, standard deviation of standard deviation; TG, training group; TINN, triangular interpolation of RR intervals; TP, total power; VLF, power in very low frequency range; VLF%, power in very low frequency range; VO_2_ max, maximum oxygen consumption; VO_2_ peak, peak oxygen consumption; WR, waist-to hip-ratio; α1, short-term component of detrended fluctuation analysis; and α2, long-term component of detrended fluctuation analysis.

**Table 2:** Outcome of heart rate-related parameters, secondary health factors, TESTEX, and STARD_HRV_ score.

Author, Year	Heart Rate-Related Parameters	Secondary Health Factors	TESTEX	STARD_HRV_
[3]	E: ↑ CI, HF, and LF; ↓ LF/HF, RHR, and SDNN. R: ↑ CI, HF, LF, and SDNN; ↓ LF/HF, and RHR. ER: ↑ LF/HF; ↔ RHR; ↓ CI, HF, LF, and SDNN,		9	20
[55]	E: ↑ ln HF; ↔ ln LF; ↓ RHR* and α1. R: ↔ α1; ↓ ln HF, ln LF, and RHR. ER: ↑ ln HF; ↓ ln LF, RHR*, and α1*	↑ VO_2_ max (E* and ER*); ↔ VO_2_ max (R)	7.5	20
[56]	↑ HF nu* and RHR; ↓ CV*, LF nu*, and LF/HF*, mRR, NN20*, NN50, pNN20, pNN50*, RMSSD, SDSD, SDNN*, TP, and VLF	↑ BF and VO_2_ max*; ↓ BM and BMI	9	18
[57]	↑ RRTri, SD1*, SD2, SD1/SD2, α1*, and α1/α2*; ↓ DFA total, TINN, and α2		6	19
[58]	LI and HI: ↑ ln LF/HF and TP (LI*); ↓ HF%, LF% and RHR*	LI and HI: ↑ HRR*; ↓ SBP (rest*, 40 W*, 80 W and 120 W)	5	19.5
[59]	↑ mRR* and RSA*; ↓ RHR*	↑ VO_2_ max*; ↓ BF*, BM*, BMI*, BR*, DBP, SBP* and WR	8	16.5
[60]	↑ HF, HF%, LF/HF, LF%, and VLF/HF; ↔ RR; ↓ LF, MSSD, TP, VLF, VLF/LF, and VLF%	↓ BM* and BMI*; ↑ VO_2_ max*	4.5	15
[61]	↑ LF nu, LF/HF, and SampEn; ↔ ln LF and ln TP; ↓ HF nu, ln HF, and RHR	↑ SBP; ↓ DBP	7	20

Abbreviations: BM, body mass; BMI, body mass index; BR, baroreflex sensitivity; CI, complexity index; CV, coefficient of variation; DBP, diastolic blood pressure; DFA, detrended fluctuation analysis; E, endurance training group; ER, endurance and resistance training group; HF (nu), power in high frequency range (in normalized units); HF%, relative power in high frequency range; HI, high intensity training; HRR, heart rate recovery; LF (nu), power in low frequency range (in normalized units); LF%, relative power in low frequency range; LI, low intensity training; ln, natural logarithm; mRR, mean RR interval; MSSD, mean squared successive differences; NN20, number of pairs of adjacent NN intervals differing by more than 20 ms in the entire recording; NN50, number of pairs of adjacent NN intervals differing by more than 50 ms in the entire recording; pNN20, NN20 count divided by the total number of all NN intervals; pNN50, NN50 count divided by the total number of all NN intervals; R, resistance training group; RHR, resting heart rate; RMSSD, square root of the mean of the sum of the squares of differences between adjacent NN intervals; RRTri, triangular index; RSA, respiratory sinus arrhythmia; SampEn, sample entropy; SBP, systolic blood pressure; SD1, standard deviation of instantaneous beat-to-beat variability extracted from Poincaré Plot; SD2, standard deviation of the long-term variability extracted from Poincaré Plot; SDNN, standard deviation of NN intervals; SDSD, standard deviation of standard deviation; TG, training group; TINN, triangular interpolation of RR intervals; TP, total power; VLF, power in very low frequency range; VLF%, power in very low frequency range; VO_2_ max, maximum oxygen consumption; VO_2_ peak, peak oxygen consumption; WR, waist-to-hip ratio; α1, short-term component of detrended fluctuation analysis; and α2, long-term component of detrended fluctuation analysis. *, significant difference between pre and post.

## Supplementary Materials

The following are available online at https://www.mdpi.com/article/10.3390/jcdd8080094/s1, Table S1: Description TESTEX, Table S2: Description STARD_HRV_, Table S2: Detailed description of the characteristics of the studies, Table S4: Results TESTEX, Table S5: Results STARD_HRV_.

## Abbreviations

ANS, autonomic nervous system; BF, body fat; BM, body mass; BMI, body mass index; BP, blood pressure; BR, baroreflex function; CI, complexity index; CV, coefficient of variation; DBP, diastolic blood pressure; ECG, electrocardiography; HDL, high density lipoprotein; HF, power in high frequency range (0.15–0.40 Hz); HRR, heart rate recovery; HRV, heart rate variability; LF, power in high frequency range (0.04–0.14 Hz); NN50, number of pairs of adjacent NN intervals differing by more than 50 ms in the entire recording; nu, normalized units; pNN50, NN50 count divided by the total number of all NN intervals; RHR, resting heart rate; RMSSD, square root of the mean of the sum of the squares of differences between adjacent NN intervals; RSA, respiratory sinus arrhythmia; SampEn, sample entropy; SBP, systolic blood pressure; SDNN, standard deviation of NN intervals; TP, total power; VO2 max, maximum oxygen consumption; VO2 peak, peak oxygen consumption; and WR, waist-to-hip ratio.
